# Promethium: To Strive, to Seek, to Find and Not to Yield

**DOI:** 10.3389/fchem.2020.00588

**Published:** 2020-07-10

**Authors:** Veronika Elkina, Mikhail Kurushkin

**Affiliations:** ^1^School #197, Saint Petersburg, Russia; ^2^Chemistry Education Research and Practice Laboratory, SCAMT Institute, ITMO University, Saint Petersburg, Russia

**Keywords:** power sources, portable x-ray sources, lasers, healthcare, illumination, measurements

## Abstract

Promethium (Pm), element #61, got its name from the Greek Titan Prometheus, who stole fire from Zeus and passed it to people. The only element in the lanthanide series of the periodic table with no stable isotopes, Pm has found an impressive number of applications since its announcement in 1947 after World War II. Despite promethium having 38 known isotopes, ^147^Pm is by far the most utilized and useful one. Promethium is used in long-life atomic batteries for satellites or space probes, satellite-to-submarine laser communication systems, “cosmic clocks” for the measurement of cosmic rays lifetime, monitoring of the changes in water content of citrus leaves caused by wetting and drying cycles in the soil, radiotherapy, and even for prevention of dandruff, to name but a few applications. During the Moon expeditions, Pm was used to illuminate instruments in the Apollo landing modules; currently it is used during preparations for long-term interplanetary missions (e.g., Mars) to simulate space conditions on Earth. This mini review offers a comprehensive illustration of promethium's history, synthesis techniques, properties, and its major applications in science, technology, and everyday life.

**Graphical Abstract:**
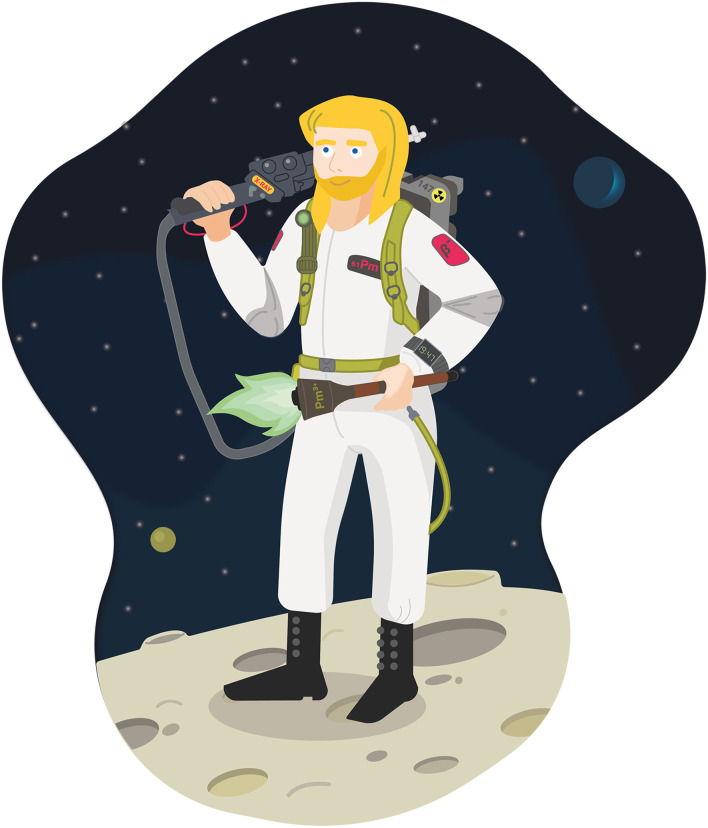
The hero of our mini review, spaceman Prometheus, with different accessories representing the versatility of promethium the element.

## Introduction

Element #61 was originally called “prometheum”, at the suggestion of the wife of one of its discoverers, in honor of the mythical hero Prometheus (Promethium, the New Name for Element 61, 1948), who stole fire from Zeus and passed it to people (Greenwood and Earnshaw, 1997a). The name was meant to emphasize not only the method of obtaining an element using nuclear fission energy, but also the threat of punishment to the instigators of war. According to Greek mythology, Zeus punished Prometheus by chaining him to a rock to be routinely tortured by an eagle (Cantrill, 2018). In 1950, the International Atomic Balance Commission gave element #61 its modern name “promethium,” while all the old names, illinium (Harris et al., 1926), florence, cyclonium, and prometheum, were rejected.

Promethium (Pm) is well-known as the only element in the lanthanide series of the periodic table with no stable isotopes (Burke, 2019); it occurs in the Earth's crust in only tiny amounts in some uranium ores. It undergoes radioactive decay of two types: electron capture and negative beta emission (Greenwood and Earnshaw, 1997b). All the promethium, which might once have existed on the Earth when it formed, would have vanished within 10,000 years.

## Synthetic Promethium

Promethium, the final lanthanide to be added to the periodic table, acquired its undeniable evidence of existence in 1945 (a discovery which was not made public until 1947) by USA chemists Jacob Marinsky, Lawrence Glendenin and Charles Coryell (Guillaumont, 2019), who isolated the radioactive isotopes ^147^Pm and ^149^Pm from uranium fission products at Clinton Laboratories (TN, USA). The thermal neutron-induced fission of ^235^U has a ^147^Pm cumulative yield (CY) of 2.25% (England and Rider, 1994). This means for every 100 fissions of ^235^U, there are 2.25 atoms of ^147^Pm produced. The ^149^Pm CY for thermal neutron-induced fission of ^235^U is only 1.08%. Ion-exchange chromatography was later utilized in order to convincingly establish Pm's identification (McGill, 2000).

Key promethium isotopes are given in Table 1; their applications will be described throughout the main body of the present mini review.

**Table 1:** Key promethium isotopes.

**Isotope**	**Year of discovery**	**Author**	**Laboratory**	**References**	**Half-life**	**Main decay mode (Nuclear Data Center at KAERI Table of Nuclides, 2020)**	**Specific activity, TBq/g (Code of Federal Regulations Title 49, 2009, Transportation, Pt. 100-185, page 656, 2009)**
^142^Pm	1959	I. Gratot	Orsay	Gratot et al., 1959	40.5(5) s	e+	N/A
^143^Pm	1952	V. Kistiakowsky	Berkeley	Kistiakowsky, 1952	265(7) d	EC	1.3 × 10^2^
^144^Pm	1952	V. Kistiakowsky	Berkeley		363(14) d	EC	9.2 × 10^1^
^147^Pm	1947	J.A. Marinsky	Oak Ridge	Marinsky et al., 1947	2.6234(2) yr	β-	3.4 × 10^1^
^149^Pm	1947	J.A. Marinsky	Oak Ridge		53.08(5) h	β-	1.5 × 10^4^

To date, 38 different isotopes of Pm are known, ranging in half-life from <1 μs to 17.7(4) years (^145^Pm) (May and Thoennessen, 2012; McLennan, 2018). For a detailed description of promethium isotopes that have been discovered to date, see (May and Thoennessen, 2012).

Fission products in nuclear fuel used to normally be the main source of ^147^Pm (Broderick et al., 2019). Until the 1970s, Oak Ridge National Laboratory was rich with ^147^Pm that had been obtained through a traditional method at Hanford, Washington (McLennan, 2018). Today, the Oak Ridge National Laboratory inventory is no longer storing promethium as the processing of nuclear fuel in the USA has been stopped, and there are no substantial sources of ^147^Pm at present. Nevertheless, identically to ^155^Eu and ^171^Tm, there is an opportunity to produce ^147^Pm by neutron capture of ^146^Nd to ^147^Nd, which β-decays to ^147^Pm, through β^−^ decay of ^147^Nd, its predecessor with a shorter half-life of nearly 11 d (Knapp, 2008).

## Main Physical and Chemical Properties

In 1974, metallic promethium was reduced from promethium oxide with metallic thorium at 1,600°C with further distillation of promethium into a quartz dome. Using this method, the melting temperature and the phase transformation temperature of promethium were established: 1042 ± 5°C and 890 ± 5°C, respectively (Angelini and Adair, 1976). Promethium's boiling point is ~3,000°C (McLennan, 2018).

The ionic radius of promethium is 110 pm (in 8-fold coordination), which is very similar to its neighboring elements, neodymium (112 pm) and samarium (108 pm) (McLennan, 2018). Hence, the close similarity in ionic radii and the same common oxidation state (+3) make it difficult to separate Pm from Nd and Sm (Balaram, 2019). When no stable isotopes exist, deductions regarding chemical properties may be drawn from known chemical surrogates (in case of promethium, other rare earth elements) (Radiological Contamination of the Oceans: Oversight Hearings Before the Subcommittee on Energy and the Environment of the Committee on Interior and Insular Affairs and House of Representatives, Ninety-fourth Congress, Second Session on Matters Pertaining to, 1976). The main Pm^3+^ compounds include: Pm(OH)_3_ (light brown), Pm_2_O_3_ (yellow white), PmCl_3_ (yellow), Pm(NO_3_)_2_ (pink), PmF_3_, Pm_2_(C_2_O_4_)_3_·10H_2_O and Pm_2_(SO_4_)_3_ (Da and Jincheng, 2000; Sharma, 2001). Pm can also represent an oxidation state of +2. Thermodynamic properties of Pm^2+^ indicate that, similarly to NdCl_2_ and SmCl_2_, stable PmF_2_, PmCl_2_ and PmI_2_ can also be obtained (Sharma, 2001).

## Power Sources

Promethium-147 is used in long-life atomic batteries (Flicker et al., 1964), in which small-scale promethium samples are inserted into a semiconductor matrix to transform their beta emission into electricity (Matheson, 1975). The mean beta energy of ^147^Pm is 62 keV (Shao et al., 2017). Pm batteries can be used in cases where other kinds of batteries would be extremely heavyweight, e.g., satellites or space probes (Vl, 1956). Radioisotope batteries are usually either thermoelectric (containing Pu or Am) (Wiss et al., 2017) based on the heat generated from radioactive decay, or betavoltaic (alphavoltaic) based on electron/hole pair generation in a semiconductor (such as ^147^Pm or other isotopes like tritium or ^63^Ni) (Gale et al., 1975; Purdy, 1986; Spencer and Chandrashekhar, 2013; Murphy et al., 2019; Xue et al., 2019). Betavoltaic batteries, compared with thermoelectric batteries (Matheson, 1975), are characterized by a smaller size and a more reasonable price. Betavoltaics also have the characteristic of lower power or current (than thermoelectric or even Li-ion batteries for example) (Gale et al., 1975; Chandrashekhar et al., 2006, 2007; Olsen et al., 2012; Murphy et al., 2019). They are useful when low power is needed for periods of years. Unfortunately, their service period does not presently exceed ten years. Newest advances in the technology of betavoltaics, however, are expected to prolong the service period to fifteen years. For example, Betacel®, a betavoltaic battery, satisfies both corrosion and cremation fire standards and is suitable for clinical use (Spencer and Chandrashekhar, 2012) and in cardiac pacemakers (Smith et al., 1975; Purdy, 1986). Promethium-147 powered microbatteries with a lifetime of up to 5 years and an average power density of 5 mW/cm^3^ are suitable candidates for implantable pacemakers (Gasper and Fester, 1975; Rosenkranz, 1975; Duggirala et al., 2007), where useful electrical power is converted from isotopic decay energy (Wheelwright and Fuqua, 1975; Greatbatch, 1980).

Despite its vast application in betavoltaic batteries, promethium can also be used in radioisotope thermoelectric generators to provide electricity for space probes (Choppin et al., 2013). Finally, promethium has also found its use as a direct lightly shielded isotopic heat source (Fullam and Van Tuyl, 1969; McNeilly and Roberts, 1969).

## Portable X-ray Sources

Although promethium-147 has low gamma emission (Artun, 2017), it is a source of soft β-rays (Malson et al., 1980). Irradiation of heavy elements with β-particles generates X-ray radiation (Ellis-Davies et al., 1985; Labrecque et al., 1986), hence, promethium must be handled strictly according to safety regulations. X-ray radiation is generated when a particular beta emitter, ^147^Pm (Sumiya et al., 1993; Llasat et al., 2017), interacts with certain d-elements like cobalt, iridium, rhodium, platinum, nickel, gold, and mixtures thereof. Radiation sources typically consist of a substrate having a non-radioactive metal surface, a metal layer of a radioactive isotope ^147^Pm, and a non-radioactive metal with a high atomic number.

## Measurements

Based on promethium-147, a commonly used energy beta source, sensors have been developed that can measure films as thin as 2.54–5.08 μm (Sneller, 1979; Brown and Coats, 1981). For instance, Adaptive Technologies Industries, Inc. (ATI) offers a modern technique based on solid-state digital beta gauge, which allows achieving real-time measurements. In ATI gauges, β-particles attenuation is employed for thickness or mass measurement of materials including plastics, paper and metal. A radiation source and a radiation detector are the two main constituents of an ATI gauge. A bulk sample of Pm is placed above the investigated material and a detector is placed below. The detector counts the amount of radiation that passes through the material. If the metal sheet becomes too thin, more radiation passes through. The technique is also employed for coat and basis weight measurements (Typpo, 2000; How beta gauge works, 2019).

Promethium-147 as a radiation source is also used to determine the thickness of sour orange and sweet lime citrus leaves that are 10–40 mg/cm^2^ thick. Interestingly, this β-ray gauging technique can also measure the changes in water content of leaves caused by wetting and drying cycles that occur in the soil (Bielorai, 1968). Alternatively, the isotopes ^14^C and ^204^Tl have also been used for various leaves mass thickness measurements (Takechi and Furudoi, 1970; Saini and Rathore, 1983). The attenuation of β-radiation from ^147^Pm can be used in miniature probes for real-time measurements of dust suspension in the 0.1–2.0 kg/m^3^ concentration range (Slezak and Buckius, 1983). Moreover, promethium-147 is used as an ionization source in electron-capture detectors for analyzing pesticides in water environments (Lubkowitz and Parker, 1971).

Another application of promethium as a pure electron-capture detector is the measurement of the mean confinement time of cosmic rays before their escape from the Galaxy (i.e., their lifetime), which is an important parameter in evaluating the sources and propagation of cosmic rays within the Galaxy. It is measured by comparing the cosmic-ray abundances of several Tc and Pm isotopes to those of neighboring, stable isotopes. The radioactive isotopes, which are most useful (^143^Pm and ^144^Pm) in “cosmic clocks,” are those with decay times comparable to the confinement time (Drach and Salamon, 1987).

## Lasers

Pm is applied in lasers that are used to communicate with submerged submarines (satellite-to-submarine laser communication systems or simply SLC). The fluorescence spectrum of Pm^3+^ is dominated by the transitions at nominally 933 and 1098 nm (Krupke et al., 1987), respectively. At room temperature, these manifolds are thermally unoccupied, a fact that enables four-level laser action at T ≈ 295 K. The high efficiency of Pm lasers and operation at 919 nm make the Pm^3+^ ion suitable for use in fully solid-state SLC laser transmitters (Shinn et al., 1988). Solid-state promethium lasers have been reported to be pumped by 2-D diode arrays operating at 770 nm (McShea et al., 1988).

## Illumination

Self-luminous sources of light for LCD watches that include a promethium-containing fluorescent layer are among the most widespread (Takami, 1980). Promethium, being usually found in the oxidized form, is not detrimental for the phosphor lattice and the material's luminosity decreases relatively slowly (Takami and Matsuzawa, 1981). Moreover, paints based on promethium isotopes, having a half-life about more than 2 years, are safer than radium alternatives. Promethium-147 is widely used not only as night lighting devices, but also as self-sustaining light sources by activating zinc sulfide phosphor with β-radiation of ^147^Pm (Ravi et al., 2001). Another use of Pm is in phosphors for highlighting various labels without energy consumption. After the discovery of radioactivity, radium acted in this capacity until its harm was revealed. Promethium compounds, however, turned out to be harmless radioactive phosphors (Rafi and Rosli, 2018). Therefore, promethium found its place in fluorescent paints. The promethium compounds used to make the characteristic “medium spring green” (pale blue-green) (Emsley, 2011) glow are usually Pm_2_O_3_ or Pm(OH)_3_ (Takami and Matsuzawa, 1981; Ravi et al., 2001; Rafi and Rosli, 2018). For example, promethium was used to illuminate instruments in the Apollo landing modules during the Moon expeditions (English et al., 1973).

## Healthcare

Sealed ^147^Pm does not represent danger due to being easily shielded (Drumheller, 1968); contrariwise, improperly stored promethium becomes an environmental hazard.

The effect of promethium intake has been vastly studied on animals, including rats, rabbits, pigs, and dogs. When absorbed by rats, promethium is predominantly retained in the bones as well as in the tips of the villi of the distal small intestine of the gastrointestinal tract, with half the dose remaining a week after probing (Sullivan et al., 1984). More recent experiments on rats' skin illustrated the ways of the radionuclides penetration (Kassai et al., 2003). To identify the penetration of Pm^3+^ ions into the cell membrane, as well as the extracellular and cellular distribution of promethium, a study was conducted on the smooth muscle of the rabbit aorta. In the course of the study, it was found that significant amounts of promethium do not accumulate inside and are not excreted from the cells, but its distribution is properly described by desorption from fibers accessible from the surface (Weiss, 1996). When pig skin is exposed to surface doses of promethium (up to 10 krads), β-particles do not affect the nature of the dose dependence of the parameters of the epidermal basal cells (Zavialov et al., 1977). When absorbed by pigs, it has been shown that most of promethium is retained in the bones similarly to the results observed in case of rats (Sullivan et al., 1984). Five and a half months after beagles were exposed to Pm_2_O_3_ aerosols, promethium was found in the organs of dogs mainly in the lungs (44%), as well as in the skeleton (24%) and in the liver (22%) (Stuart, 1966).

Surprisingly, since early 80s little has been discovered regarding the effect promethium has on human organs; however, bone tissues are possible candidates (Metabolic data for promethium, 1981). Promethium-147 can be identified and analyzed in urine and feces using a simple co-precipitation technique, which applies mainly to the excrements of former employees of promethium processing plants (Berk and Moghissi, 1985). In the case of inhalation of promethium-containing luminous paints, most of it settles in the lungs, practically not excreted. A few days after inhalation due to phagocytosis, the activity is observed as “hotspots” in macrophages in bronchial epithelium and alveolar walls, mostly at the periphery of the lung lobes (Kraus, 1976). If swallowed, promethium-147 passes through the digestive tract without being absorbed into the walls of the lower large intestine; radiation doses can be measured by examining human feces (Vennart, 1967).

In medicine, promethium beta therapy can cure lumbosacral radiculitis (Purdy, 1986). At a Geneva hospital, ^142^Pm was used in an *in vivo* generator for preclinical positron emission tomography (Beyer and Ruth, 2003). Promethium-149, in turn, as a medium-energy beta emitter, is a suitable radilolanthanide for receptor-targeted radiotherapy (Studer et al., 2019). A great advantage of ^149^Pm is its low intensity emission of imageable γ-rays (286 keV), which provides *in vivo* tracking of the therapeutic dose (Hu et al., 2002).

Furthermore, promethium can prevent hair loss, promote hair regrowth and black hair formation as well as remove or even prevent dandruff (Kim and Choi, 2014).

## Conclusions, Outlook, and Outer Space

Here, we have summarized the history, synthesis techniques and the major applications of promethium. Although peak interest in Pm was in the 1980's, it has recently been receiving renewed attention: for instance, promethium is featured among strategic materials in the 2013 model year Ford Fiesta, Focus, Fusion and F-150 (Field et al., 2017).

The future research on Pm is expected to bring us into the outer space. Promethium is used as a prototype radiation source in attempts to simulate space conditions on Earth (Hellweg et al., 2007). Since cosmic radiation is identified as the most hazardous for the health of crew participating in long-term interplanetary missions (e.g., Mars), ^147^Pm radiation is used in biological experiments aimed at determination of the allowed irradiation dose range of human embryonic kidney (HEK) cells survival (Hellweg et al., 2008).

In 2004, the possible identification of Pm in the spectra of HD 965 and HD 101065 was reported (Cowley et al., 2004). Recognition was based on statistical and traditional line-identification methods (Fivet et al., 2007). Promethium is also occasionally found as few atoms from uranium decay detected in the HR 465 star spectrum of Andromeda. The star is evidently manufacturing Pm on its surface, taking into account that no Pm isotope with a half-life longer than that of ^145^Pm can exist. Thus, the elusive origin of Pm in outer space is yet to be explained (Emsley, 2011).

## Author Contributions

VE was responsible for literature search and analysis and initial draft preparation. MK was responsible for formulating the mini review objectives and finalizing the initial draft. Both authors contributed to the article and approved the submitted version.

## Conflict of Interest

The authors declare that the research was conducted in the absence of any commercial or financial relationships that could be construed as a potential conflict of interest.
